# Endoscopic Removal of Sinonasal Inverted Papilloma Originating From the Posterior Ethmoid Cavity

**DOI:** 10.1155/crot/8389174

**Published:** 2025-02-20

**Authors:** Sofia E. Olsson, René Peña

**Affiliations:** ^1^Department of Otolaryngology, Anne Burnett Marion School of Medicine, Texas Christian University, 2800 South University Dr, Fort Worth 76109, Texas, USA; ^2^Department of Otolaryngology, Central Park ENT, 409 Central Park Dr., Arlington 76014, Texas, USA

**Keywords:** inverted papilloma, nasal mass, nasal obstruction, sinus surgery

## Abstract

Sinonasal inverted papilloma is an expansive, benign mass derived from the Schneiderian membrane. It may undergo malignant transformation and most commonly originates from the maxillary sinuses or the lateral walls of the nasal corridors. This case outlines the case of a sinonasal inverted papilloma, which clearly arises from the posterior ethmoid sinus, bordering the skull base. This abnormal originating point was able to be identified during endoscopic excision of the mass and involved mucosa. The ethmoid bone was not resected as it would expose the dura mater, risking CSF leak and complications. This case further supports the use of endoscopy in the investigation of sinonasal inverted papilloma rather than the gold standard approach of lateral rhinotomy. An endoscopic approach allowed for improved safety when accessing the posterior ethmoid cavity. This case also highlights the possibility of novel origins of sinonasal inverted papilloma, such as the membrane of the posterior ethmoid cavity.

## 1. Introduction

Inverted papilloma is a sinonasal neoplasm derived from the Schneiderian respiratory membrane [1]. They most commonly present unilaterally with symptoms of nasal obstruction, epistaxis, or pain [1]. Sinonasal papillomas have the potential for malignant transformation at variable rates estimated to be up to 11% [2, 3]. Surgical resection is necessary to prevent malignant transformation, but recurrence is possible at rates estimated up to 25.3% [3–6].

First described in 1854, sinonasal inverted papillomas currently have an annual incidence of 0.2–1.5 cases per 100,000 people, representing 0.5%–4% of all sinonasal neoplasms [7–9]. The etiology of this neoplasm is idiopathic with proposed causes including human papilloma virus infection, Epstein–Barr virus infection, allergies, chronic inflammation, and nicotine usage [1, 8].

Sinonasal inverted papillomas primarily originate at the lateral nasal walls or maxillary sinuses, arising from the Schneiderian membrane [10]. In this report, we discuss a sinonasal inverted papilloma with a unique origin of the posterior ethmoid cavity bordering the sphenoid sinus along with the endoscopic surgical approach used for its resection.

## 2. Case Presentation

**Figure 1:**
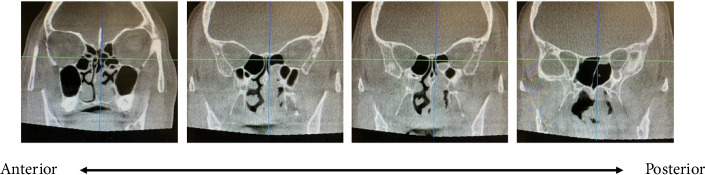
Computed tomographic scan of nasal mass prior to endoscopic resection.

A 59-year-old female with a past medical history of hypertension controlled with losartan and osteoporosis presented with 1 year of worsening nasal congestion, pressure, and parosmia. Her obstructive symptoms were more noticeable on the left. Over-the-counter oral antihistamines, nasal steroids, and sinonasal rinsing provided minimal relief. Anterior rhinoscopy revealed a severe right-sided deviated septum with an enlarged left middle turbinate. The nasal mucosa appeared pink and moist with no discharge, crusting, malformations, or foreign bodies. Rigid nasal endoscopy revealed an estimated 3-mm tip of a papillomatous mass filling the mid left nasal cavity. The origin of the mass could not be identified, and its course was unable to be tracked via rigid nasal endoscopy; however, nasal obstruction due to enlargement of the middle turbinate was evident. Computed tomography (CT) scan showed a soft tissue density filling the left midnasal cavity with a single opacified posterior ethmoid cell on the left side (Figure 1). The patient was fully informed of all treatment options including surgery versus observation with medical management. She decided to proceed with surgical excision with septoplasty and possible left ethmoidectomy. The patient consented to the sharing of her anonymized case as a published case report.

**Figure 2:**
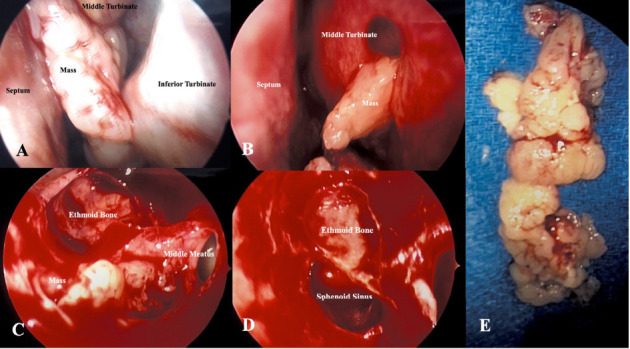
Endoscopic view of (A) mass obstructing nasal cavity, (B) mass emerging from the middle meatus, (C) the mass following medialization of the middle meatus, (D) location of mass origin following resection, and (E) postoperative view of mass.

Image-guided endoscopic endonasal excision of the left sinonasal mass was performed with left total ethmoidectomy and septoplasty (Figures 2(A), 2(B), 2(C), 2(D), and 2(E)). The mass appeared to pierce through the left middle turbinate into the middle portion of the nasal cavity (Figure 2(B)) with posterior extension to the to the choana and posterior ethmoid cavity. The mass was removed in pieces from within the nasal cavity with the anterior portion being removed with large straight forceps. Anterior ethmoid cells were widely opened with microdebridement and the mass was followed to the stalk, which appeared to be confined to a single posterior ethmoid cell. The remainder of the mas along with the mucosa of the posterior ethmoid cavity that encompassed the stalk was completely removed. The sphenoid sinus ostium also was widely opened using the microdebrider. Pathology confirmed a sinonasal papilloma, inverted type. There was no evidence of malignancy or dysplasia and human papillomavirus typification was not performed.

The patient followed up in clinic 5 days after surgery where sinonasal rigid endoscopy with debridement was performed. The mucosa was healing well, and the patient reported relief of her symptoms with no complaints upon removal of intranasal packing. The patient has been under follow-up for a total of 4 months with complete symptom resolution. She will be monitored for tumor recurrence for at least five postoperative years with annual follow-up visits and nasal endoscopy.

## 3. Discussion

The current gold standard treatment protocol for sinonasal inverted papilloma is aggressive lateral rhinotomy and medial maxillectomy [10]. Historically, midfacial degloving has been used as an alternative approach for extensive lesions [10]. With the rise of endoscopic sinus surgery, surgeons more frequently attempt papilloma resection via an endoscopic approach or a combined approach depending on lesion location and extent of involvement [10]. If invasion of the papilloma into surrounding structures is severe or endoscopy does not confidently remove all involved structures, open surgery must be considered.

A vital step in reducing recurrence rate is ensuring complete removal of the mass and involved mucosa. Beginning with an endoscopic approach rather than open surgery in this case allowed for tracking of the papilloma to the mucosal surface of the posterior superior ethmoid cavity, bordering the sphenoid sinuses and the sella. This is an uncommon anatomic location for sinonasal inverted papillomas to originate with limited representation in the related literature [3, 11]. Endoscopic surgery is the preferred approach to ethmoidectomy, with open approaches rarely used in cases of uncomplicated tumor involvement [12].

Due to variability of the aggressive nature of sinonasal papillomas, the patient must be monitored for several years postoperatively, and wider open resection may be considered in the case of disease recurrence. Most commonly, recurrence will occur within a year of resection in the same anatomic location; however, 20% recur five or more years after resection [13]. Early on in the tumor recurrence, patients may present with nonspecific symptoms such as congestion or rhinorrhea. Due to the high recurrence rates and lack of symptoms, patients must be monitored routinely for recurrence with endoscopy or magnetic resonance imaging for at least 5 years after resection [14].

## 4. Conclusion

Inverted papillomas are a relatively rare benign neoplasm, which often presents with years of nasal obstruction. They tend to arise from the Schneiderian membrane of the lateral nasal wall and maxillary sinuses; however, identifying their anatomic origin is vital in preventing recurrence or malignant transformation. This case of a patient with an inverted papilloma originating from the left posterior ethmoid and tracking through the left middle turbinate highlights the necessity of visualizing the origin and excising all involved tissue, as well as the benefit to utilizing an endoscopic surgical approach to widely open the sinus cavities, allowing for optimal postoperative surveillance for any recurrence.

## Data Availability

The data that support the findings of this study are available on request from the corresponding author. The data are not publicly available due to privacy or ethical restrictions.
